# Constraint-Based Modeling of Carbon Fixation and the Energetics of Electron Transfer in *Geobacter metallireducens*

**DOI:** 10.1371/journal.pcbi.1003575

**Published:** 2014-04-24

**Authors:** Adam M. Feist, Harish Nagarajan, Amelia-Elena Rotaru, Pier-Luc Tremblay, Tian Zhang, Kelly P. Nevin, Derek R. Lovley, Karsten Zengler

**Affiliations:** 1 Department of Bioengineering, University of California San Diego, La Jolla, California, United States of America; 2 Department of Microbiology, University of Massachusetts, Amherst, Massachusetts, United States of America; The Pennsylvania State University, United States of America

## Abstract

*Geobacter* species are of great interest for environmental and biotechnology applications as they can carry out direct electron transfer to insoluble metals or other microorganisms and have the ability to assimilate inorganic carbon. Here, we report on the capability and key enabling metabolic machinery of *Geobacter metallireducens* GS-15 to carry out CO_2_ fixation and direct electron transfer to iron. An updated metabolic reconstruction was generated, growth screens on targeted conditions of interest were performed, and constraint-based analysis was utilized to characterize and evaluate critical pathways and reactions in *G. metallireducens*. The novel capability of *G. metallireducens* to grow autotrophically with formate and Fe(III) was predicted and subsequently validated *in vivo*. Additionally, the energetic cost of transferring electrons to an external electron acceptor was determined through analysis of growth experiments carried out using three different electron acceptors (Fe(III), nitrate, and fumarate) by systematically isolating and examining different parts of the electron transport chain. The updated reconstruction will serve as a knowledgebase for understanding and engineering *Geobacter* and similar species.

## Author Summary

The ability of microorganisms to exchange electrons directly with their environment has large implications for our knowledge of industrial and environmental processes. For decades, it has been known that microbes can use electrodes as electron acceptors in microbial fuel cell settings. *Geobacter metallireducens* has been one of the model organisms for characterizing microbe-electrode interactions as well as environmental processes such as bioremediation. Here, we significantly expand the knowledge of metabolism and energetics of this model organism by employing constraint-based metabolic modeling. Through this analysis, we build the metabolic pathways necessary for carbon fixation, a desirable property for industrial chemical production. We further discover a novel growth condition which enables the characterization of autotrophic (i.e., carbon-fixing) metabolism in *Geobacter*. Importantly, our systems-level modeling approach helped elucidate the key metabolic pathways and the energetic cost associated with extracellular electron transfer. This model can be applied to characterize and engineer the metabolism and electron transfer capabilities of *Geobacter* for biotechnological applications.

## Introduction

Microorganisms play a major role in the global carbon cycle. Insights into the various mechanisms and energetic constrains which govern their behavior will advance our understanding of carbon fluxes and might ultimately allow for a rational perturbation of the carbon cycle. Key features of the carbon cycle are the conversion of organic and inorganic carbon and the energy flow through the system. Qualitative and quantitative knowledge of carbon assimilation and electron flow to and from key microorganisms is critical when evaluating certain aspects of the carbon cycle. Members of the genus *Geobacter* are ubiquitous in the soil environment, and have been described to utilize various organic substrates while transferring electrons to insoluble metals externally [1]. Furthermore, certain *Geobacter* species, such as *G. metallireducens* have been reported to transfer electrons directly to poised electrodes [2] and even to other microbes [3], a process coined direct interspecies electron transfer or DIET. Quantitative assessment of carbon and energy flow in *G. metallireducens* by computational modeling approaches therefore provides valuable insight into the role of this bacterium in the carbon cycle.

*Geobacter metallireducens*, the first *Geobacter* species that was isolated [4], serves as a pure culture model for the study of many of the important reactions that *Geobacter* species catalyze in the biogeochemistry of anaerobic soils and sediments, groundwater bioremediation, and several bioenergy applications [5]. For example, *Geobacter* species play a major role in the biogeochemical cycling due to their ability to couple the oxidation of organic compounds to the reduction of Fe(III) and Mn(IV) oxides [5]. *G. metallireducens* was the first microorganism shown to be capable of the anaerobic degradation of aromatic hydrocarbons [6] and Fe(III) is an important electron acceptor for the removal of aromatic hydrocarbons in many contaminated subsurface environments [5]. Direct electron transfer from electrodes to microorganisms to drive anaerobic respiration has potential applications in bioenergy and bioremediation [7].

Constraint-based reconstruction and analysis (COBRA) is a powerful method for characterizing the content of an organism, or systems of organisms, and understanding the limits of its collective functionality [8]. Metabolic network reconstruction (the most widely utilized form of COBRA) enables the enumeration of the genome-wide machinery (i.e., enzymes, uptake systems, etc.) in an organized fashion for use in modeling [9]. With a reconstructed network for an organism, predictions can be made about its functionality when combined with physiological data in a modeling framework. Further, a validated and accurate network can be utilized for prospective design and engineering of cellular networks [10].

There is a history of modeling *Geobacter sp.* using COBRA [11]. One of the first studies utilizing COBRA and *Geobacter* was for *G. sulfurreducens* [12]. The key findings of this study were an initial reconstruction and examination of the extracellular electron transport, the examination of the efficiency of internal biomass biosynthetic pathways, and predictions of gene deletion phenotypes. A subsequent study branched off to build a reconstruction of *G. metallireducens* based on the original content of the *G. sulfurreducens* reconstruction [13]. The initial *G. metallireducens* reconstruction was used to examine the efficiency of pathway usage in the network along with yield on a variety of substrates.

In this work, an updated reconstruction was built and analyzed to better understand key capabilities of *G. metallireducens*. The updated reconstruction effort was fueled by the appearance of a more complete genome annotation [14] and new data available for the electron transport chain and key metabolic content [15], [16].

## Results/Discussion

### Construction, comparison, and validation of the *Geobacter metallireducens* GS-15 genome-scale metabolic reconstruction

**Figure 1:**
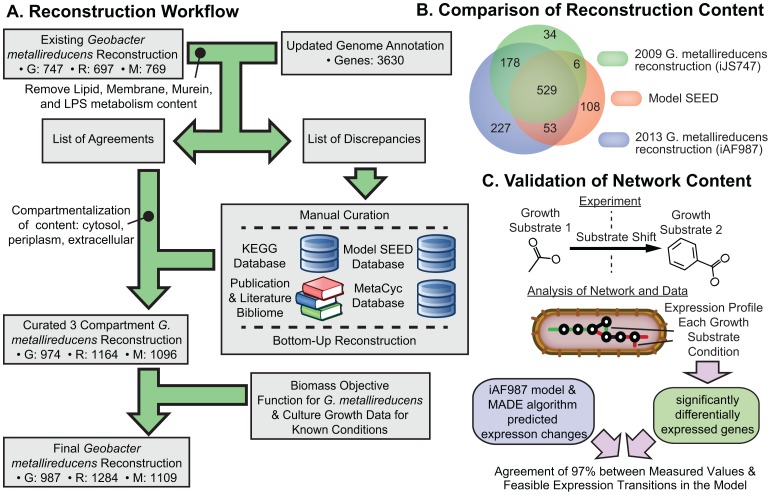
The workflow developed to generate iAF987 along with comparison and validation of its content. A) The workflow detailing the reconstruction process of the *G. metallireducens* metabolic network. The reconstruction process was initiated by comparing the updated genome annotation for *G. metallireducens* to the existing reconstruction to create a list of discrepancies that was manually reviewed and curated. Content that was in agreement with the updated annotation and reconstruction was used to generate a draft set of intracellular reactions. Lipid, membrane, murein, and LPS content were removed from this list as a periplasm compartment was added to the reconstruction. Manual curation [9] was aided by the KEGG [46], ModelSEED [20], and MetaCyc [35] databases. Further, numerous publications and literature sources (i.e., the “bibliome”) were used to refine the network content. The manual review process resulted in a draft reconstruction that was used in conjunction with a formulated biomass objective function in simulations to validate the content of the reconstruction and generate a final version. Some figure images adapted from [47]. B) Venn Diagram showing the comparative analysis of gene content included in different versions of a *G. metallireducens* reconstruction. C) A schematic of the validation of network content with transcriptomics data for a shift from acetate growth conditions to benzoate with the MADE computational algorithm.

An updated reconstruction of *G. metallireducens* GS-15, iAF987, was generated by reconciling an existing genome-scale reconstruction [13] and an updated genome annotation, performing a bottom-up reconstruction of additional metabolic pathways. This new reconstruction was functionally tested for performance under known growth conditions (Figure 1A). The final reconstruction contained 987 genes, 1284 reactions, and 1109 metabolites. In the first phase, the existing reconstruction was compared to the updated genome annotation [14] to identify a list of agreements, discrepancies, and scope for expansion. A distinct periplasm compartment was determined to be important as *G. metallireducens* has the unique ability to transfer electrons extracellularly [5]. Thus, characterizing the electron transfer pathways from the cytosol through the periplasm to the extracellular space was crucial for understanding this unique capability. Furthermore, the addition of the periplasm compartment allows for a more accurate representation of metabolism, such as *p*-cresol and 4-hydroxybenzyl alcohol degradation, which partially occurs in the periplasm [17]. A wild-type and a reduced ‘core’ biomass objective function [18] were formulated to validate whether the reconstruction could generate the appropriate biomass components necessary to replicate, and for use in simulation to predict the growth rates of the organism on the different substrates. Gaps were filled in the network using data characterizing growth of *G. metallireducens* GS-15 on 19 different carbon sources/electron donors with Fe(III) as the electron acceptor, and the SMILEY algorithm [19] (see Text S1).

The iAF987 reconstruction was compared to the previous version [13] and an automatically generated reconstruction from the ModelSEED framework [20] (Figure 1B) was used to identify and evaluate newly reconstructed and unique content. The ModelSEED reconstruction was found to have 114 unique genes that were not present in the iAF987 reconstruction. Of these, 86 genes were involved in macromolecular synthesis, DNA replication, and protein modifications that are beyond the scope of a metabolic network, and 8 of them did not have a specific reaction association in the ModelSEED (i.e., generic terms such as aminopeptidase, amidohydrolase). Of the remaining 20 genes, only two (Gmet_0988 and Gmet_2683) were added to the reconstruction as isozymes for existing reactions; the other 18 assignments conflicted with our functional annotation of the genome and thus were not included. The iAF987 reconstruction contains 227 genes not in either reconstruction, thus representing a significant advancement of coverage. These newly included genes encode several unique pathways, encoding 325 unique reactions, which have not previously appeared in a collection of 14 representative reconstructions (Table 1) from the UCSD database from which iAF987 was constructed and is internally consistent (see Text S1 for a detailed comparison of iAF987 to previous work).

**Table 1:** Subsystem distribution of reactions unique to the *G. metallireducens* iAF987 reconstruction.

Subsystem	Number of Reactions
Transport	75
Lipids and Glycan metabolism	64
Vitamins & Cofactor Biosynthesis	51
Aromatic Compound degradation	26
Alternate Carbon Metabolism	21
Amino Acid Metabolism	18
Energy Metabolism	17
Central Metabolism	15
Nitrogen and Sulfur Metabolism	12
Nucleotide Metabolism	11
Other	9
Metal Respiration	6
**Total**	**325**

Transcriptomic data profiling a growth shift from acetate to the aromatic compound benzoate was integrated with the metabolic model to validate its content. Specifically, the computational analysis was performed using the MADE algorithm [21] which uses the statistical significance of changes in gene expression to create a functional metabolic model that most accurately recapitulates the expression dynamics. Of the 987 genes in the metabolic model, transcriptomic data indicated that the expressions of 857 genes do not change significantly and 130 were differentially expressed (>2-fold and p-value<0.05). Specifically, 77 genes were up-regulated and 53 were down-regulated during this shift. The MADE algorithm predicted that during this metabolic shift, the expression of 885 genes in iAF987 do not change significantly and 102 genes are differentially expressed. Of the 102 differentially expressed genes, the MADE algorithm predicted the up-regulation of 70 genes and down-regulation of 32 genes. Specifically, the model predicted upregulation of 70 genes, while data indicated 77 genes to be upregulated during this shift. Similarly while the model predicted downregulation of 32 genes, the data actually indicated that 53 genes were downregulated during this shift. The model-based prediction of change in expression disagreed with the *in vitro* transcriptomic data for only 28 of the 987 genes leading to 97% overall agreement (for a more detailed breakdown, see Text S1 and Table S1). Among the genes differentially expressed during the shift, the genes encoding for benzoyl-CoA reductase were up-regulated over 100-fold during benzoate growth. It was determined that this key enzyme that links the degradation of aromatic substrates to central metabolism is not ATP driven as previously thought [13], but is likely membrane bound and proton translocating [16]. Thus, a proton translocating reaction was added to the reconstruction for this step in metabolism. A translocation stoichiometry of 3 protons per electron was determined to be the likely extent of coupling through a thermodynamic analysis (see Text S1). Similar transcriptomic analyses for growth shifts on two other aromatic electron donors (i.e., toluene and phenol) yielded 86% and 84% agreement, respectively (Table S1). These findings will likely broaden our knowledge of how *G. metallireducens* can be utilized for bioremediation.

### Analysis of unique metabolic capabilities: Carbon fixation and external electron transfer pathways

**Figure 2:**
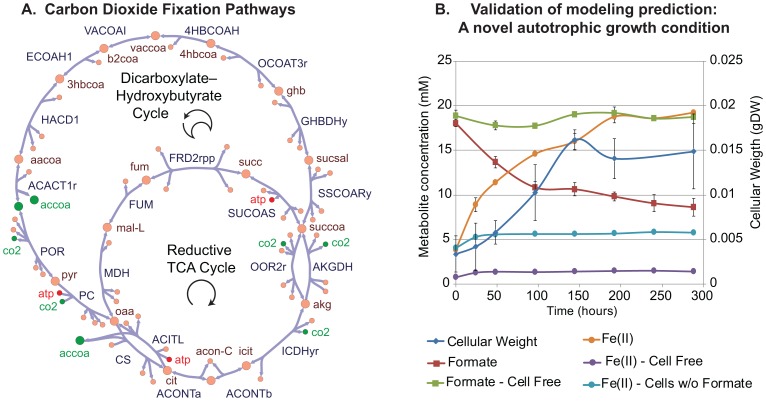
Reconstruction, analysis, and wet lab validation of carbon fixation pathways in *G. metallireducens*. A) A map of the two carbon fixation pathways included in the iAF987 reconstruction and encoded by genes annotated in the updated genome annotation. The two pathways, the reductive citric acid (TCA), and the dicarboxylate–hydroxybutyrate cycles share four reactions in the citric acid cycle. CO_2_ fixation (and resulting acetyl-coA generated) and ATP-driven steps are shown in green and red, respectively. B) A graph of phenotypic data that demonstrated the model-predicted growth condition of formate as an electron donor and carbon source with Fe(III) as an electron acceptor is a viable growth condition. Carbon fixation is occurring under these conditions and the specifics of this process can be further elucidated in subsequent “drill-down” studies.

The genome of *G. metallireducens* GS-15 encodes two out of the six known carbon fixation pathways [22]. The pathways which were reconstructed in iAF987 are the reductive citric acid cycle (rTCA) and the dicarboxylate–hydroxybutyrate cycle [22] (Figure 2A). Key enzymes for the rTCA include the 2-oxoglutarate synthase (abbreviated OOR2r in the reconstruction) and ATP-citrate lyase (ACITL), both of which enable the citric acid cycle to run in reverse. For the dicarboxylate–hydroxybutyrate cycle, the key enzyme is 4-hydroxybutyryl-CoA dehydratase (4HBCOAH). The rTCA and the dicarboxylate–hydroxybutyrate cycles share four reactions. The reconstruction of these carbon fixation pathways led to the prediction of a new growth condition for *G. metallireducens* (Table 2). With the expanded content including the carbon fixation pathways, it was computationally predicted that *G. metallireducens* can grow with formate as the electron donor and Fe(III) as the electron acceptor when a computational screen of all possible media combinations was performed with the model (Table 2). Investigating the resulting flux distributions revealed that the CO_2_ derived from formate oxidation is reduced via the rTCA to form acetyl-CoA which is subsequently assimilated into biomass. The electrons derived from formate oxidation are split between running the rTCA and for Fe(III) reduction. The energy gained by formation of a proton gradient during Fe(III) reduction was instrumental for providing the required ATP for carbon fixation. This prediction of growth on formate and Fe(III) was experimentally validated, Figure 2B. The requirement of CO_2_ fixation for growth of *G. metallireducens* solely on formate and CO_2_ as carbon sources is further highlighted by a study which examined *G. sulfurreducens* reducing Fe(III) with formate as the electron donor. While *G. sulfurreducen*s was able to reduce Fe(III) with formate as electron donor, it required the addition of 0.1 mM acetate to assimilate cell carbon (i.e., grow) [23]. This was attributed to the lack of an rTCA in *G. sulfurreducens*, specifically due to the absence of the ATP-dependent citrate lyase. Overall, this example represents a unique power of the model to rapidly generate hypotheses *in silico* that can then be verified experimentally. This particular result proves to be of great interest for examining carbon fixation.

**Table 2:** Validated and predicted carbon sources and electron donors for *G. metallireducens*.

Experimentally and Computationally Validated Carbon Sources and Electron Donors	Computationally Predicted Carbon Sources and Electron Donors	Experimentally and Computationally Validated Electron Acceptors
3-Methylbutanoic acid, p-Cresol, 4-Hydroxy-benzyl alcohol, 4-Hydroxybenzaldehyde, 4-Hydroxybenzoate, Acetate, Butanol, Butyrate (n-C4:0), Benzoate, Benzaldehyde, Benzyl alcohol, Ethanol, Isobutyrate, Phenol, Propionate (n-C3:0), Propanol, Pentanoate, Pyruvate, Toluene	Formate†, Glycerol, Long Chain Fatty Acids (Saturated and Unsaturated)	Fe(III), Manganese Mn(IV), Uranium U(VI), Technetium Tc(VII), Vanadium V(V), Nitrate, Nitrite

† Formate was validated as a carbon source and electron donor experimentally in this study.

### Examining the cost of external electron transfer

**Figure 3:**
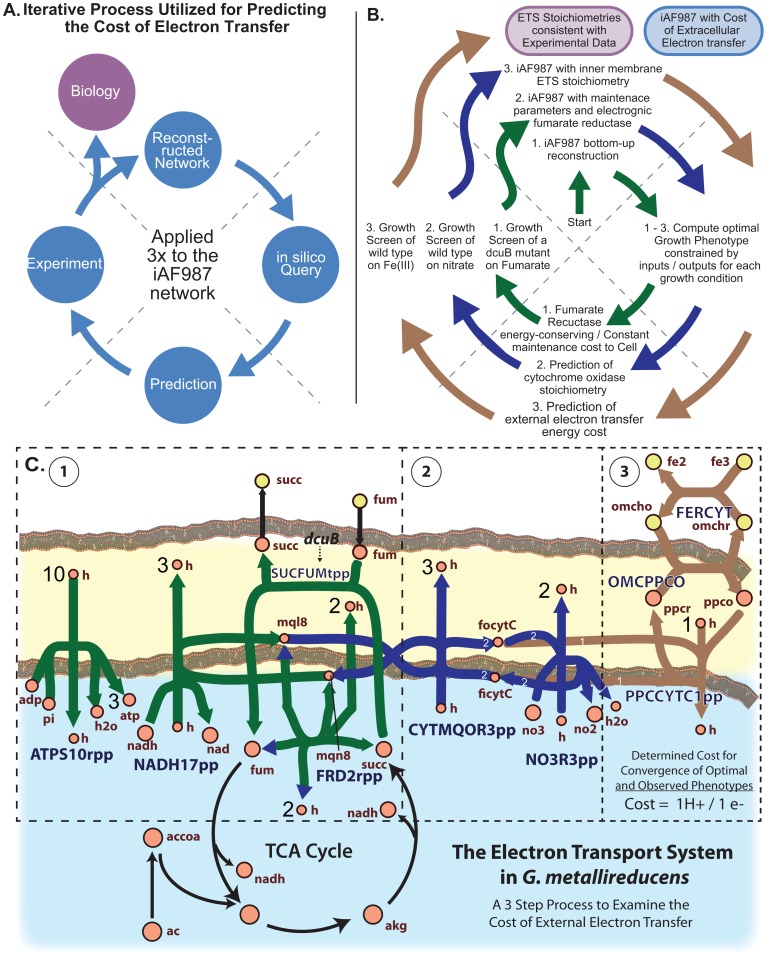
A model-driven analysis of the electron transport system in *G. metallireducens*. A) schematic of the iterative loop process common to model-driven analyses, and (B) the process applied to examine the cost of the electron transport system in *G. metallireducens*. C) Map of the updated ETS in the iAF987 reconstruction. The functional states of the network components during internal electron transfer to fumarate are shown in green (facilitated by the *dcuB* strain), additional components active during internal transfer to nitrate are shown in blue, and additional components during external electron transfer (Fe respiration) are shown in brown. Note that the fumarate reductase (FRD2rpp) operates in opposite directions depending on whether the electron acceptor is fumarate or nitrate/Fe(III). Also note that nitrate reductase (NO3R3pp) is not used when Fe(III) is the electron acceptor. Abbreviations are defined in Dataset S2. The process was started with the bottom-up reconstruction of the updated metabolic network. At each cycle around the loop, optimal performance was calculated as different components of the ETS were isolated, compared to experimental data, and then incorporated into the reconstruction. Ultimately, the final product is a reconstructed ETS consistent with experimental data and an estimate of the cost associated with transferring electrons from the internal membrane cytochrome (focytC) to the extracellular electron acceptor Fe(III) (fe3).

To reconstruct the electron transport system of *G. metallireducens*, key steps involving electron transfer to the terminal electron acceptor were subject to a thermodynamic analysis (Text S1). Specifically, the feasibility of proton translocation and the theoretical maximum proton translocation stoichiometry was determined [24], [25]. Subsequently, the included content was analyzed with physiological data from growth screens to predict stoichiometries for the key reactions of the electron transport system (ETS). This was performed in a model-driven iterative process with an approach that delineates the energetics of extracellular electron transfer by examining three distinct modules (Figure 3A–B). These modules were characterized by representative electron acceptors; fumarate, nitrate, and Fe(III), respectively. Reduction of fumarate in *G. metallireducens* requires the strain to harbor the *dcuB* gene [26]; nitrate is reduced via the ETS and the nitrate reductase [14], [27]; extracellular Fe(III) requires several extracellular *c*-type cytochromes and pili [28], which have been shown in the closely related *G. sulfurreducens* to have metal-like conductivity [29]. Acetate consumption has previously been described as a preferred optimal growth condition for *G. metallireducens* [27], therefore it was used as the primary electron donor when analyzing the ETS in this study. The pathways and key ETS reactions for this conversion are shown in Figure 3C and evidence for the inclusion of the reactions in iAF987 is given in the Text S1.

The first step in the modeling process was to examine the fumarate reductase reaction and maintenance energies necessary for predicting phenotypes using constraint-based analysis (Step 1, Figure 3C). To examine this content, data was generated using the *G. metallireducens* dcuB strain and utilized in simulations. An electrogenic bifunctional fumarate reductase/succinate dehydrogenase (FRD2rpp) was included based on recent biochemical evidence in a similar species [15]. This inclusion was validated with the growth data of *G. metallireducens* on acetate and fumarate using the dcuB strain. The earlier version of the *G. metallireducens* model [13] and the current model (iAF987) without an electrogenic fumarate reductase were unable to produce a feasible solution when constrained with experimentally measured acetate and fumarate uptake rates (see Table 3). However, the electrogenic fumarate reductase enabled the model to reproduce the experimental observations and was assigned to translocate two protons per two electrons transferred to fumarate. It was determined that two protons were necessary to drive the endergonic oxidation of succinate with menaquinol as part of the TCA cycle during Fe(III) respiration and nitrate reduction (see Text S1). Maintenance energies were also evaluated in this step by choosing a growth condition that eliminated any use of the external electron transferring reactions (so these reactions could be examined in isolation later). *G. metallireducens* dcuB strain was grown in batch using acetate/fumarate medium and also in a chemostat at a set growth rate (Table 3). The growth rate determined in this study was similar to that previously reported (0.114 hr^−1^ vs 0.105 hr^−1^, respectively) [26]. From these two conditions, it was possible to estimate the maintenance costs (GAM and NGAM) for the model using an established procedure [9], [18]. The calculated costs were 79.20 mmol ATP gDW^−1^ for the GAM and 0.81 mmol ATP gDW^−1^ hr^−1^ for the NGAM. These values are similar to those found previously for *G. sulfurreducens* [12], thus it provided confidence in the use of the GAM and NGAM throughout the study. The additional content included in the ETS was further analyzed using experimental data.

**Table 3:** Phenomic and modeling data from growth screens of *G. metallireducens* GS-15 wild type and dcuB with acetate as an electron donor.

Strain	Acceptor/Growth Mode (Donor Acetate)	Growth Rate (hr-1)	Donor Uptake Rate (mmol gDW-1 hr-1)	Acceptor Uptake Rate (mmol gDW-1 hr-1)	Acceptor/Donor Ratio
**GS-15 ** ***dcuB***	Fumarate/Batch	0.114±0.005	6.81±0.10	14.0±0.2	2.06±0.01
**GS-15 ** ***dcuB***	Fumarate/Chemostat*	0.05	2.79±0.23	N.D.	N.D.
**GS-15**	Nitrate/Batch∧ (byproduct ammonia)	0.111±0.022	5.20±2.08	4.67±0.64 (no3) −3.98±0.38 (nh4)	0.90±0.42
**GS-15**	Fe(III)/Batch	N.D.	N.D.	N.D.	6.96±0.43
**GS-15**	Fe(III)/Chemostat*	0.05	7.86±1.02	58.29±9.08	7.41±0.64
**Simulation GS-15 ** ***dcuB***	Max Growth Rate Fumarate	0.116	6.22	14.2 (lim)	2.28
**Simulation GS-15 ** ***dcuB***	Max Growth Rate Fumarate	0.054	3.02 (lim)	7.06	2.34
**Simulation GS-15**	Max Growth Rate Nitrate	0.118	7.28 (lim)	4.35 −3.61 (lim)	0.60
**Simulation GS-15**	Max Growth Rate Fe(III)	0.047	8.88 (lim)	62.31	7.02

* Donor-limited chemostat;

N.D., Not Determined; lim, limiting rate in the simulation;

∧ calculated error using a 90% confidence interval from Lovley and Phillips, 1988 [27].

The second step in the modeling process to examine extracellular transfer was to examine the energetics of the menaquinone cytochrome oxidoreductase reaction (CYTMQOR3) in the ETS (Step 2, Figure 3B). This was performed by switching the electron acceptor from fumarate to nitrate, while keeping acetate as the electron donor. Assuming the nitrate reductase translocates two protons per two electrons (a value that has been verified [30]), the translocation stoichiometry of the menaquinone cytochrome oxidoreductase can be isolated. Analyzing the energetics of the CYTMQOR3 reaction, it was determined that a likely number of three protons are translocated per two electrons transferred to the cytochrome pool (see Text S1). Using this stoichiometry and simulating growth with acetate and limiting nitrate (see Table 3), the predicted optimal growth rate was calculated to be 0.054 hr^−1^ as compared to the experimentally determined value of 0.050 hr^−1^ (in this experiment, the growth rate is equal to the set dilution rate in a steady state chemostat). Thus, it was concluded that the proton translocating assignment of the CYTMQOR3 reaction was consistent with the experimental results of the growth screen.

The final step taken to predict the energetic cost of transferring electrons to an external substrate was to examine growth of *G. metallireducens* on acetate and Fe(III) after reconciling the other components of the ETS consistent with observed phenotypic data. Figure 3C, step 3 shows the reconstructed path from the inner membrane cytochromes to the outer membrane cytochromes and eventually to reduce Fe(III). An energetic cost was estimated in terms of an ATP cost proportional to the flux of electrons to Fe(III). Growth screens of wild type *G. metallireducens* (acetate/Fe(III)) were performed in triplicate in both batch and chemostat cultures. When culturing with Fe(III) as an electron acceptor, a measurement of biomass is challenging given that the optical density is used to calculate the amount of reduced iron (see Methods). Therefore, the ratio of acceptor produced to donor consumed was used to compare to simulations, as it is more precise than a biomass-normalized uptake and production rate that was calculated by measuring protein content in the chemostat. Furthermore, the ratio of acceptor to donor calculated for both the batch and chemostat conditions was very similar, thus providing a consistent experimental comparison (see Table 3). A Phenotypic Phase Plane (PhPP) analysis [31] was used to compare model-predicted performance to the experimentally measured acceptor to donor ratio. It was calculated that a cost of one proton translocated across the inner membrane per one electron transferred ultimately to Fe(III) best matched the line of optimality in the PhPP analysis (see Figure 3 C, Figure S1). Further, this cost is very close to 0.3 ATP per electron transferred ultimately to Fe(III), as the ATP synthase in the cell converts protons to ATP at a ratio of 3.33 protons per ATP (see Text S1). Further analysis of this modeling approach with phenotypic data on different electron donors (butanol, ethanol, and pyruvate) yielded the same cost of external electron transfer (see Table S3). Thus, it was hypothesized that this is the approximate cost for external electron transfer to iron and the reactions and costs were built into the iAF987 reconstruction as such. This cost can now be further validated for different external electron transfer processes that *G. metallireducens* is known to carry out.

### Conclusion

The work presented here demonstrates how constraint-based modeling and reconstruction can be applied to generate hypotheses that can be tested experimentally. Specifically, modeling revealed a non-obvious culturing condition where carbon fixation could be directly examined. Further, the cost of external electron transfer could be quantified using an iterative and systematic approach. Carbon sequestration is of great biotechnological interest [32]. By understanding the mode of growth for CO_2_ fixation, computational predictions can be used to guide genetic modifications which enhance the rate of CO_2_ fixation. Specifically, this could be in the form of reaction knockouts or identification of genes which could be targeted for overexpression which are predicted to enhance CO_2_ fixation. The reconstructed model advances our knowledge for this unique species and provides a platform for further analysis and hypothesis formulation for environmental and biotechnology applications.

## Methods

### Updated annotation

Curation of the genome annotation of *G. metallireducens* was continued after the initial publication [14] with additional insights from curation of the genome annotations of *Geobacter bemidjiensis* [33], *Pelobacter carbinolicus* [34], and other species (M. Aklujkar, unpublished), with extensive reference to the MetaCyc database [35]. The updated annotation was submitted to NCBI with reference numbers CP000148 and CP000149.

### Reconstruction process

The reconstruction was generated in a four-step process. First, the updated genome annotation for *G. metallireducens* was entered into the UCSD SimPheny (Genomatica, San Diego, CA) database [14]. Next, the existing *G. metallireducens* reconstruction [13] was mapped to the updated genome using the existing gene-protein-reaction associations [8] and all of the pathways excluding membrane lipid biosynthesis, lipopolysaccharide biosynthesis, murein biosynthesis and degradation, and transport were entered into the SimPheny framework if an exact match for the reaction was present in the UCSD SimPheny database. If an exact match for the reaction did not exist in the UCSD SimPheny database on the level of metabolites participating in the reaction, they were manually evaluated for inclusion (see below). Next, a comparison of the metabolic content included in the updated genome annotation (Dataset S1) that was not in SimPheny was performed. Manual evaluation of new content or disagreements from the annotation and existing genome-scale reconstruction consisted of gathering genetic, biochemical, sequence, and physiological data and reconciling this information to determine the likelihood of each reaction being present in the organism. This manual curation process has been described and reviewed several times [8], [9]. In the manual review process, the KEGG database (www.genome.jp/kegg/), the ModelSEED database [20], and primary literature (see Dataset S2) were used extensively in the manual curation process. Confidence scores were given for each reaction along with noteworthy evidence used to justify inclusion of a given reaction.

#### Reconciling the reconstruction content with ModelSEED

The gene content in the *G. metallireducens* reconstruction was compared and reconciled with the automated reconstruction obtained from ModelSEED [20]. The ModelSEED reconstruction was found to have 119 unique genes that were not present in the *G. metallireducens* reconstruction. Upon further analysis, it was determined that 86 of these genes were involved in macromolecular synthesis, DNA replication, and protein modifications that are beyond the scope of a metabolic network. Of the remaining 33 genes, eight did not have a specific reaction association in the ModelSEED (i.e., generic terms such as aminopeptidase, amidohydrolase). The ModelSEED annotations of the 25 metabolic genes were compared with the updated genome annotation presented in this work. It was found that these two sets of annotations were consistent for only two genes (*G. metallireducens*et_0988 and *G. metallireducens*et_2683). These two genes were added to the reconstruction by associating them to the appropriate reaction. In the case of the 23 genes where a discrepancy existed between the updated genome annotation and ModelSEED annotation, the updated annotation was taken as gold standard. No reactions were added or removed from the reconstruction as a result of this analysis.

### Generation of the Biomass Objective Function (BOF)

The BOFs were formulated using a previous template [36] and are included in Dataset S3. The biomass content previously determined for the close species *Geobacter sulfurreducens* was used to determine the breakdown of macromolecules [12] except that for total carbohydrate as the distribution in the murein, lipopolysaccharide, and cytosolic fractions was not indicated. Further, the genome annotation [14] was used for the breakdown of chromosome bases, a study on lipid and lipopolysaccharide chain length was used for the breakdown of acyl chain length [37], and the remaining content was approximated using the full profile presented for the gram-negative bacterium *E. coli* [36]. It should be noted that prediction of growth rate and unmeasured uptake rates are relatively insensitive realistic variations in biomass macromolecular weight fractions [36]. The BOF components included in the core BOF were extrapolated from the core BOF formulated for *E. coli* as Geobacter sp. have the same gram-negative cell morphology.

### Flux balance analysis simulations

The COBRA Toolbox 2.0 [38] and the SimPheny framework (Genomatica, Inc., San Diego, CA) were used for simulations. Constraints used to simulate growth in ferric citrate medium are presented in Text S1. For the evaluation of the maintenance energies, the best-fit values of acetate and fumarate were set to the exact values.

### *In vivo* growth screens

Growth screens were performed in triplicate using cultures in 125 mL serum bottles under anoxic conditions using Fe(III) or fumarate (with the *dcuB* mutant studies) as an electron acceptor. The composition of the fumarate medium and ferric citrate medium were the same as previously described [39] and [40], respectively. The concentrations of the electron donors utilized in the experiments were 15 mM acetate, 20 mM ethanol, and 10 mM butanol. For the growth screens, the cells were passed in the media in which they were being tested for at least three passages before the growth screen was performed. For the dcuB mutant strain, the correlation between OD and biomass that was used was 0.4561 gDW L-1 OD600-1. This value was determined by growing the *dcuB* cells in freshwater medium, taking OD measurements at various time points, and weighing the dried biomass at various points of the growth curve. The cells were dried overnight in an oven and weighed on filter paper, with a correction for the amount of weight lost by a filter paper that did not contain cells. Analytes were quantified by HPLC using an Aminex 87-H ion exchange column at 65° C. The mobile phase was 5 mM H_2_SO_4_ at an isocratic flow of 0.5 mL/minute. Sample injection volume was 10 µL. Products were identified by retention time using ultraviolet detection at 210 nm and refractive index detection at 30° C internal temperature and 45° C external temperature and quantified by relating peak area to those of standards. Fe(II) was measured using the ferrozine assay as described [40]. Growth rates were calculated by determining the exponential growth phase region from a series of samples taken for each growth screen (typically, 7–10 samples were taken over the entire screen). For this corresponding exponential growth phase region, the ratio of an analyte to the gDW of the sample was determined using a linear fit obtained through the least-squares method (‘regress’ function in MATLAB). This value (mmol gDW-1) was then multiplied by the growth rate to get a corresponding uptake or production rate. The ratios were subsequently multiplied by the growth rate to get the uptake or production rates. Averages and standard deviations were reported and calculated from three biological replicates for each experiment. For the *G. metallireducens* GS-15 dcuB stain, the fumarate and succinate rates were independently calculated and then averaged for each replicate as there is a 1∶1 ratio for the dcuB exchange and experimental accuracy for each analyte differed slightly. The fumarate uptake rate was calculated using the net fumarate and malate concentrations measured [41]. Malate was observed when growing with ethanol and butanol as electron donors with the *dcuB* strain. For the acceptor rates in these two conditions, the succinate production rate was used as the rate of the donor to minimize compounding measurement error (although the effective fumarate uptake rate was very similar, <27% difference in any individual replicate).

Chemostat cultures of *G. metallireducens* GS-15 wild-type and *dcuB* cultures were grown at 30°C in anaerobic continuous culture vessels as previously described [42]. The GS-15 wild-type strain was grown under donor limiting conditions with 5 mM acetate and 55 mM ferric citrate, the *dcuB* strain was grown under acetate limiting conditions with 5 mM Acetate and 27.5 mM fumarate. A protein conversion value of protein content (ug/mL) = 0.4344*dry-cell-weight (ug/mL) was used to calculate rates.

To determine if *G. metallireducens* was capable of growth with formate, cells were adapted to 20 mM formate in a ferric-citrate medium [40] and not provided with any other electron donor. After the third transfer with formate as the sole electron donor, samples were withdrawn anaerobically and substrate consumption was monitored. Formate was detected via high performance liquid chromatography. Reduction of Fe(III) to Fe(II) was monitored with the ferrozine assay. Experiments were run with triplicate cultures. The number of cells was monitored over time until the cultures reached a plateau of Fe(II) production. To fix cells, 900 µL culture was withdrawn anaerobically and mixed with 100 µL glutaraldehyde (25%). Fixed cells were stored at −20°C until processed. All samples were defrosted, placed on filters, filtered and washed with sterile water prior to staining with acridin orange for 3 minutes. Filters were washed and dried. Cell counts were carried out for three biological samples by manually counting at least 5 microscopic fields (Area 5826 µm^2^ per field) per sample, with the help of a cell counting application in ImageJ (http://rsbweb.nih.gov/ij/). Control cultures consisting of (i) cells in ferric-citrate medium with no formate, and (ii) ferric-citrate medium with formate and no cells (i.e., cell-free) were also performed.

### Transcriptomic analysis

For the microarray experiments performed, *G. metallireducens* was grown anaerobically with Fe(III) citrate (55 mM) as the electron acceptor and one of the following electron donors: acetate (10 mM), benzoate (1 mM), phenol (0.5 mM), or toluene (0.5 mM). Cells were grown in 1 L bottles and harvested during mid-exponential phase by centrifugation. The cell pellet was immediately frozen in liquid nitrogen and stored at −80 °C. RNA was isolated from triplicate cultures grown on each electron donor with a modification of the previously described method [43]. Briefly, cell pellets were resuspended in HG extraction buffer [44] pre-heated at 65 °C. The suspension was incubated for 10 minutes at 65 °C to lyse the cells. Nucleic acids were isolated with a phenol-chloroform extraction followed by ethanol precipitation. The pellet was washed twice with 70% ethanol, dried, and resuspended in sterile diethylpyrocarbonate-treated water. RNA was then purified with the RNA Clean-Up kit (Qiagen, Valencia, CA, USA) and treated with DNA-free DNASE (Ambion, Woodward, TX, USA). The RNA samples were tested for genomic DNA contamination by PCR amplification of the 16S rRNA gene. cDNA was generated with the TransPlex Whole Transcriptome Amplification Kit (Sigma).

Whole-genome microarray hybridizations were carried out by Roche NimbleGen, Inc. (Madison, WI, USA). Triplicate biological and technical replicates were conducted for all microarray analyses. Cy3-labeled cDNA was hybridized to oligonucleotide microarrays based on the *G. metallireducens* genome and resident plasmid sequences (accession number NC007515 and NC007517 at GenBank). All the microarray data has been deposited with NCBI GEO under accession number GSE33794.

For each metabolic shift (benzoate vs acetate, toluene vs acetate, phenol), the fold-changes in expression level and p-value (t-test) were computed using ArrayStar 4.02 (DNASTAR, Madison, WI, USA). This was used in conjunction with the iAF987 metabolic model to predict the metabolic adjustment using the MADE algorithm [21]. Additionally, the constraints for the iAF987 metabolic model were set to simulate growth on the respective substrates. MADE analysis was implemented using the TIGER toolbox [45]. The output of this analysis consisted of the genes predicted by MADE algorithm to significantly change in expression during the concerned metabolic shift.

## Supporting Information

Dataset S1 An Excel file containing the updated genome annotation for *G. metallireducens*.
(XLSX)

Dataset S2 An Excel file containing the content of the iAF987.
(XLSX)

Dataset S3 The biomass objective function formulated for *G. metallireducens* and a table corresponding to changes which were made to the network based on functional testing using the biomass objective function.
(XLSX)

Dataset S4 SBML files of the iAF987 reconstruction.
(ZIP)

Dataset S5 Transcriptomic data for *G. metallireducens* on acetate, benzoate, toluene, and phenol as electron donors and Fe(III) as the electron acceptor.
(XLSX)

Figure S1 A Phenotypic Phase Plane (PhPP) analysis to approximate the energetic cost of external electron transfer. Modeling was used to estimate a cost of external electron transfer. After applying the cost (in the box indicated below) to the network (see Figure 3C) by making it proportional to the flux of electrons moving onto Fe(III), the line of optimality (LOO) matches the experimental value of donor uptake to Fe(III) conversation rate.
(EPS)

Figure S2 The ETS that was reconstructed in previous Geobacter reconstructions. Significant changes were made to the ETS in the iAF987 reconstruction as compared to the previously reconstructed content (see Figure 3C). The previous reconstructions and subsequent models did not account for the dissipation of membrane potential during Fe(III) respiration due to succinate oxidation by menaquinone. Instead, a net proton cost is being assumed at the menaquinone cytochrome oxidase step (also, mqn7 used instead of mqn8). The analysis performed in this study, decouples the lumped cost, and by including the appropriate stoichiometry of the electrogenic fumarate reductase is able to mechanistically distinguish between fumarate reduction and Fe(III) reduction.
(EPS)

Table S1 Results of the MADE analysis.
(PDF)

Table S2 Phenotypic and modeling data from growth screens of *G. metallireducens* GS-15 dcuB.
(PDF)

Table S3 Phenotypic and modeling data from growth screens of *G. metallireducens* GS-15 to support the estimated cost of external electron transfer.
(PDF)

Table S4 SBML files properties.
(PDF)

Table S5 The *in silico* (computational) minimal media.
(PDF)

Text S1 Supplementary text and methods.
(PDF)

## References

[1] Lovley DR, Giovannoni SJ, White DC, Champine JE, Phillips EJ, et al (1993) Geobacter metallireducens gen. nov. sp. nov., a microorganism capable of coupling the complete oxidation of organic compounds to the reduction of iron and other metals. Archives of microbiology 159: 336–344. 8387263 10.1007/BF00290916

[2] Logan BE (2009) Exoelectrogenic bacteria that power microbial fuel cells. Nature reviews Microbiology 7: 375–381. 19330018 10.1038/nrmicro2113

[3] Summers ZM, Fogarty HE, Leang C, Franks AE, Malvankar NS, et al (2010) Direct exchange of electrons within aggregates of an evolved syntrophic coculture of anaerobic bacteria. Science 330: 1413–1415. 21127257 10.1126/science.1196526

[4] Lovley DR, Stolz JF, Nord GL, Phillips EJP (1987) Anaerobic production of magnetite by a dissimilatory iron-reducing microorganism. Nature 330: 252–254.

[5] Lovley DR, Ueki T, Zhang T, Malvankar NS, Shrestha PM, et al (2011) Geobacter: the microbe electric's physiology, ecology, and practical applications. Adv Microb Physiol 59: 1–100. 22114840 10.1016/B978-0-12-387661-4.00004-5

[6] Lovley DR, Baedecker MJ, Lonergan DJ, Cozzarelli IM, Phillips EJP, et al (1989) Oxidation of aromatic contaminants coupled to microbial iron reduction. Nature 339: 297–299.

[7] Lovley DR, Nevin KP (2013) Electrobiocommodities: powering microbial production of fuels and commodity chemicals from carbon dioxide with electricity. Curr Opin Biotechnol 24: 385–390. 23465755 10.1016/j.copbio.2013.02.012

[8] Feist AM, Herrgard MJ, Thiele I, Reed JL, Palsson BO (2009) Reconstruction of biochemical networks in microorganisms. Nat Rev Microbiol 7: 129–143. 19116616 10.1038/nrmicro1949 PMC3119670

[9] Thiele I, Palsson BO (2010) A protocol for generating a high-quality genome-scale metabolic reconstruction. Nat Protoc 5: 93–121. 20057383 10.1038/nprot.2009.203 PMC3125167

[10] McCloskey D, Palsson BO, Feist AM (2013) Basic and applied uses of genome-scale metabolic network reconstructions of Escherichia coli. Molecular Systems Biology 9: 661. 23632383 10.1038/msb.2013.18 PMC3658273

[11] Mahadevan R, Palsson BO, Lovley DR (2011) In situ to in silico and back: elucidating the physiology and ecology of Geobacter spp. using genome-scale modelling. Nat Rev Microbiol 9: 39–50. 21132020 10.1038/nrmicro2456

[12] Mahadevan R, Bond DR, Butler JE, Esteve-Nunez A, Coppi MV, et al (2006) Characterization of Metabolism in the Fe(III)-Reducing Organism Geobacter sulfurreducens by Constraint-Based Modeling. Appl Environ Microbiol 72: 1558–1568. 16461711 10.1128/AEM.72.2.1558-1568.2006 PMC1392927

[13] Sun J, Sayyar B, Butler JE, Pharkya P, Fahland TR, et al (2009) Genome-scale constraint-based modeling of Geobacter metallireducens. BMC Syst Biol 3: 15. 19175927 10.1186/1752-0509-3-15 PMC2640342

[14] Aklujkar M, Krushkal J, DiBartolo G, Lapidus A, Land ML, et al (2009) The genome sequence of Geobacter metallireducens: features of metabolism, physiology and regulation common and dissimilar to Geobacter sulfurreducens. BMC Microbiol 9: 109. 19473543 10.1186/1471-2180-9-109 PMC2700814

[15] Zaunmuller T, Kelly DJ, Glockner FO, Unden G (2006) Succinate dehydrogenase functioning by a reverse redox loop mechanism and fumarate reductase in sulphate-reducing bacteria. Microbiology 152: 2443–2453. 16849807 10.1099/mic.0.28849-0

[16] Kung JW, Loffler C, Dorner K, Heintz D, Gallien S, et al (2009) Identification and characterization of the tungsten-containing class of benzoyl-coenzyme A reductases. Proceedings of the National Academy of Sciences of the United States of America 106: 17687–17692. 19815533 10.1073/pnas.0905073106 PMC2764909

[17] Johannes J, Bluschke A, Jehmlich N, von Bergen M, Boll M (2008) Purification and characterization of active-site components of the putative p-cresol methylhydroxylase membrane complex from Geobacter metallireducens. Journal of Bacteriology 190: 6493–6500. 18658262 10.1128/JB.00790-08 PMC2566014

[18] Feist AM, Palsson BO (2010) The biomass objective function. Curr Opin Microbiol 13 (3) 344–9. 20430689 10.1016/j.mib.2010.03.003 PMC2912156

[19] Reed JL, Patel TR, Chen KH, Joyce AR, Applebee MK, et al (2006) Systems approach to refining genome annotation. Proceedings of the National Academy of Sciences of the United States of America 103: 17480–17484. 17088549 10.1073/pnas.0603364103 PMC1859954

[20] Henry CS, DeJongh M, Best AA, Frybarger PM, Linsay B, et al (2010) High-throughput generation, optimization and analysis of genome-scale metabolic models. Nat Biotechnol 28: 977–982. 20802497 10.1038/nbt.1672

[21] Jensen PA, Papin JA (2011) Functional integration of a metabolic network model and expression data without arbitrary thresholding. Bioinformatics 27: 541–547. 21172910 10.1093/bioinformatics/btq702 PMC6276961

[22] Berg IA, Kockelkorn D, Ramos-Vera WH, Say RF, Zarzycki J, et al (2010) Autotrophic carbon fixation in archaea. Nature reviews Microbiology 8: 447–460. 20453874 10.1038/nrmicro2365

[23] Coppi MV, O'Neil RA, Leang C, Kaufmann F, Methé BA, et al (2007) Involvement of *Geobacter sulfurreducens* SfrAB in acetate metabolism rather than intracellular Fe(III) reduction. Microbiology 153: 3572–3585. 17906154 10.1099/mic.0.2007/006478-0

[24] Thauer RK, Jungermann K, Decker K (1977) Energy conservation in chemotrophic anaerobic bacteria. Bacteriological reviews 41: 100–180. 860983 10.1128/br.41.1.100-180.1977 PMC413997

[25] Kroger A, Biel S, Simon J, Gross R, Unden G, et al (2002) Fumarate respiration of Wolinella succinogenes: enzymology, energetics and coupling mechanism. Biochimica et biophysica acta 1553: 23–38. 11803015 10.1016/s0005-2728(01)00234-1

[26] Butler JE, Glaven RH, Esteve-Nunez A, Nunez C, Shelobolina ES, et al (2006) Genetic characterization of a single bifunctional enzyme for fumarate reduction and succinate oxidation in Geobacter sulfurreducens and engineering of fumarate reduction in Geobacter metallireducens. Journal of Bacteriology 188: 450–455. 16385034 10.1128/JB.188.2.450-455.2006 PMC1347312

[27] Lovley DR, Phillips EJ (1988) Novel mode of microbial energy metabolism: organic carbon oxidation coupled to dissimilatory reduction of iron or manganese. Applied and environmental microbiology 54: 1472–1480. 16347658 10.1128/aem.54.6.1472-1480.1988 PMC202682

[28] Smith JA, Lovley DR, Tremblay PL (2013) Outer cell surface components essential for Fe(III) oxide reduction by *Geobacter metallireducens* . Appl Environ Microbiol 79: 901–907. 23183974 10.1128/AEM.02954-12 PMC3568551

[29] Malvankar NS, Vargas M, Nevin KP, Franks AE, Leang C, et al (2011) Tunable metallic-like conductivity in nanostructured biofilms comprised of microbial nanowires. Nature Nanotechnology 6: 573–579. 10.1038/nnano.2011.119 21822253

[30] Garland PB, Downie JA, Haddock BA (1975) Proton translocation and the respiratory nitrate reductase of Escherichia coli. The Biochemical journal 152: 547–559. 5996 10.1042/bj1520547 PMC1172508

[31] Edwards JS, Ibarra RU, Palsson BO (2001) *In silico* predictions of *Escherichia coli* metabolic capabilities are consistent with experimental data. Nat Biotechnol 19: 125–130. 11175725 10.1038/84379

[32] Latif H, Zeidan AA, Nielsen AT, Zengler K (2014) Trash to treasure: production of biofuels and commodity chemicals via syngas fermenting microorganisms. Current Opinion in Biotechnology 27: 79–87. 24863900 10.1016/j.copbio.2013.12.001

[33] Aklujkar M, Young ND, Holmes D, Chavan M, Risso C, et al (2010) The genome of Geobacter bemidjiensis, exemplar for the subsurface clade of Geobacter species that predominate in Fe(III)-reducing subsurface environments. BMC Genomics 11: 490. 20828392 10.1186/1471-2164-11-490 PMC2996986

[34] Aklujkar M, Haveman SA, DiDonato R Jr, Chertkov O, Han CS, et al (2012) The genome of Pelobacter carbinolicus reveals surprising metabolic capabilities and physiological features. BMC Genomics 13: 690. 23227809 10.1186/1471-2164-13-690 PMC3543383

[35] Caspi R, Altman T, Dreher K, Fulcher CA, Subhraveti P, et al (2012) The MetaCyc database of metabolic pathways and enzymes and the BioCyc collection of pathway/genome databases. Nucleic Acids Research 40: D742–753. 22102576 10.1093/nar/gkr1014 PMC3245006

[36] Feist AM, Henry CS, Reed JL, Krummenacker M, Joyce AR, et al (2007) A genome-scale metabolic reconstruction for *Escherichia coli* K-12 MG1655 that accounts for 1260 ORFs and thermodynamic information. Mol Syst Biol 3: 121. 17593909 10.1038/msb4100155 PMC1911197

[37] Hedrick DB, Peacock AD, Lovley DR, Woodard TL, Nevin KP, et al (2009) Polar lipid fatty acids, LPS-hydroxy fatty acids, and respiratory quinones of three Geobacter strains, and variation with electron acceptor. J Ind Microbiol Biotechnol 36: 205–209. 18846396 10.1007/s10295-008-0486-7

[38] Schellenberger J, Que R, Fleming RM, Thiele I, Orth JD, et al (2011) Quantitative prediction of cellular metabolism with constraint-based models: the COBRA Toolbox v2.0. Nat Protoc 6: 1290–1307. 21886097 10.1038/nprot.2011.308 PMC3319681

[39] Lovley DR, Phillips EJP (1988) Novel mode of microbial energy metabolism: organic carbon oxidation coupled to dissimilatory reduction of iron or manganese. Appl Environ Microbiol 54: 1472–1480. 16347658 10.1128/aem.54.6.1472-1480.1988 PMC202682

[40] Butler JE, Glaven RH, Esteve-Nunez A, Nunez C, Shelobolina ES, et al (2006) Genetic characterization of a single bifunctional enzyme for fumarate reduction and succinate oxidation in *Geobacter sulfurreducens* and engineering of fumarate reduction in *Geobacter metallireducens* . J Bacteriol 188: 450–455. 16385034 10.1128/JB.188.2.450-455.2006 PMC1347312

[41] Galushko AS, Schink B (2000) Oxidation of acetate through reactions of the citric acid cycle by Geobacter sulfurreducens in pure culture and in syntrophic coculture. Archives of microbiology 174: 314–321. 11131021 10.1007/s002030000208

[42] Esteve-Nunez A, Rothermich M, Sharma M, Lovley D (2005) Growth of Geobacter sulfurreducens under nutrient-limiting conditions in continuous culture. Environmental microbiology 7: 641–648. 15819846 10.1111/j.1462-2920.2005.00731.x

[43] Holmes DE, Risso C, Smith JA, Lovley DR (2011) Anaerobic oxidation of benzene by the hyperthermophilic archaeon Ferroglobus placidus. Applied and environmental microbiology 77: 5926–5933. 21742914 10.1128/AEM.05452-11 PMC3165377

[44] Holmes DE, Risso C, Smith JA, Lovley DR (2012) Genome-scale analysis of anaerobic benzoate and phenol metabolism in the hyperthermophilic archaeon Ferroglobus placidus. The ISME journal 6: 146–157. 21776029 10.1038/ismej.2011.88 PMC3246244

[45] Jensen PA, Lutz KA, Papin JA (2011) TIGER: Toolbox for integrating genome-scale metabolic models, expression data, and transcriptional regulatory networks. BMC Syst Biol 5: 147. 21943338 10.1186/1752-0509-5-147 PMC3224351

[46] Kanehisa M, Goto S (2000) KEGG: kyoto encyclopedia of genes and genomes. Nucleic Acids Res 28: 27–30. 10592173 10.1093/nar/28.1.27 PMC102409

[47] Nagarajan H, Sahin M, Nogales J, Latif H, Lovley DR, et al (2013) Characterizing acetogenic metabolism using a genome-scale metabolic reconstruction of Clostridium ljungdahlii. Microb Cell Fact 12: 118. 24274140 10.1186/1475-2859-12-118 PMC4222884

