# Supplementary material for: Constraint-Based Modeling of Carbon Fixation and the Energetics of Electron Transfer in Geobacter metallireducens
Source: PLoS Comput Biol. 2014 Apr 24;10(4):e1003575. doi: 10.1371/journal.pcbi.1003575 (PMC3998878; doi:10.1371/journal.pcbi.1003575)
Supplement: Table S1 — Results of the MADE analysis. (PDF) [file pcbi.1003575.s008.pdf]

Table S1: Results of the MADE analysis

	Acetate / Benzoate	Acetate / Toluene	Acetate / Phenol
**Differentially expressed genes**	523	1687	1953
**Differentially expressed model genes**	130	520	590
**Upregulated genes (Data)**	77	316	285
**Downregulated genes (Data)**	53	304	305
**Upregulated genes (Model)**	70	200	258
**Downregulated genes (Model)**	32	186	179
**Percent Match**	97%	86%	84%
