# Supplementary material for: Constraint-Based Modeling of Carbon Fixation and the Energetics of Electron Transfer in Geobacter metallireducens
Source: PLoS Comput Biol. 2014 Apr 24;10(4):e1003575. doi: 10.1371/journal.pcbi.1003575 (PMC3998878; doi:10.1371/journal.pcbi.1003575)
Supplement: Table S2 — Phenotypic and modeling data from growth screens of G. metallireducens GS-15 dcuB. (PDF) [file pcbi.1003575.s009.pdf]

Table S2: Phenotypic and modeling data from growth screens of *G. metallireducens* GS-15 dcuB

Strain	Condition	Growth Rate (hr-1)	Donor Uptake Rate (mmol gDW-1 hr-1)	Acceptor Uptake Rate (mmol gDW-1 hr-1)	Acceptor / Donor Ratio
**GS-15 *dcuB***	ethanol fumarate batch	0.052 ± 0.001	-4.41 ± 0.80	-12.7 ± 0.1	2.88 ± 0.52
**GS-15 *dcuB***	butanol fumarate batch	0.024 ± 0.003	-0.59 ± 0.25	-6.01 ± 0.10	10.2 ± 4.3
**Simulation GS-15 *dcuB***	Max Growth Rate * ethanol fumarate batch	0.127	-4.09	-12.8 (lim)	3.13
**Simulation GS-15 *dcuB***	Max Growth Rate butanol fumarate batch	0.053	-0.84 (lim)	-5.22	6.21

lim, limiting rate in the simulation; * acetate excretion from the model disabled
