# Supplementary material for: Constraint-Based Modeling of Carbon Fixation and the Energetics of Electron Transfer in Geobacter metallireducens
Source: PLoS Comput Biol. 2014 Apr 24;10(4):e1003575. doi: 10.1371/journal.pcbi.1003575 (PMC3998878; doi:10.1371/journal.pcbi.1003575)
Supplement: Table S3 — Phenotypic and modeling data from growth screens of G. metallireducens GS-15 to support the estimated cost of external electron transfer. (PDF) [file pcbi.1003575.s010.pdf]

Table S3: Phenotypic and modeling data from growth screens of *G. metallireducens* GS-15 to support the estimated cost of external electron transfer

Strain	Condition Donor / Acceptor / Growth Mode	Experimental Acceptor / Donor Ratio	In silico Acceptor / Donor Ratio - No Cost	In silico Acceptor / Donor Ratio - With Cost of external electron transfer
**GS-15**	ethanol / Fe(III) / Batch	9.19 ± 0.98	6.86	9.32
**GS-15**	butanol / Fe(III) / Batch	8.50 ± 0.88 ^	7.12	8.42
**GS-15**	pyruvate / Fe(III) / Batch	8.73 ± 2.22	5.09	6.92

^ butyrate was seen as a byproduct at a byproduct to donor ratio of 0.67 ± 0.04.
