# Supplementary material for: Constraint-Based Modeling of Carbon Fixation and the Energetics of Electron Transfer in Geobacter metallireducens
Source: PLoS Comput Biol. 2014 Apr 24;10(4):e1003575. doi: 10.1371/journal.pcbi.1003575 (PMC3998878; doi:10.1371/journal.pcbi.1003575)
Supplement: Table S4 — SBML files properties. (PDF) [file pcbi.1003575.s011.pdf]

Table S4: SBML Files Properties

**SBML File Properties**	
file name	*Gm_iAF987_ac_dcuB*.xml
organism	Geobacter metallireducens GS-15
model	iAF987
Biomass Objective Function (BOF)	Gm_biomass_GS15_WT_79p20M (*G. metallireducens* biomass objective function (iAF987) - WT - with 79.20 GAM estimate)
flux balance analysis objective	Maximize WT BOF
Growth Associated Maintenance (GAM)	79.20 mmol ATP gDW-1
Non-Growth Associated Maintenance (NGAM)	0.81 mmol ATP gDW-1 hr-1
media conditions	computational minimal media
Carbon source and electron donor	6.81 +/- 0.1 mmol acetate gDW-1 hr-1
Electron acceptor	14.0 +/- 0.2 mmol fumarate gDW-1 hr-1
additional constraints	
reactions constrained to zero	HYD4pp, NAD-H2, HDR3
Reactions allowed to operate in only one direction	G3PD1, G3PD2, G3PD3
Reactions included in model for the *dcuB* mutant	EX_fum(e), EX_succ(e), FUMtex, SUCCtex, SUCFUMtpp

**SBML File Properties**	
file name	*Gm_iAF987_ac_Fe*.xml
organism	Geobacter metallireducens GS-15
model	iAF987
Biomass Objective Function (BOF)	Gm_biomass_GS15_WT_79p20M (*G. metallireducens* biomass objective function (iAF987) - WT - with 79.20 GAM estimate)
flux balance analysis objective	Maximize WT BOF
Growth Associated Maintenance (GAM)	79.20 mmol ATP gDW-1
Non-Growth Associated Maintenance (NGAM)	0.81 mmol ATP gDW-1 hr-1
media conditions	computational minimal media
Carbon source and electron donor	7.86 +/- 1.02 mmol acetate gDW-1 hr-1
Electron acceptor	58.29 +/- 9.08 mmol Fe(III) gDW-1 hr-1
additional constraints	
reactions constrained to zero	HYD4pp, NAD-H2, HDR3
Reactions allowed to operate in only one direction	G3PD1, G3PD2, G3PD3
