# Supplementary material for: Constraint-Based Modeling of Carbon Fixation and the Energetics of Electron Transfer in Geobacter metallireducens
Source: PLoS Comput Biol. 2014 Apr 24;10(4):e1003575. doi: 10.1371/journal.pcbi.1003575 (PMC3998878; doi:10.1371/journal.pcbi.1003575)
Supplement: Table S5 — The in silico (computational) minimal media. (PDF) [file pcbi.1003575.s012.pdf]

Table S5: The *in silico* (computational) minimal media

Metabolite Name	Metabolite Abbreviation	Excahnge Reaction	Reaction Equation	Note
calcium	ca2	EX_ca2(e)	[e] : ca2 <==>	ion
cadmium	cd2	EX_cd2(e)	[e] : cd2 <==>	ion
chloride	cl	EX_cl(e)	[e] : cl <==>	ion
carbon dioxide	co2	EX_co2(e)	[e] : co2 <==>	other
cobalt (2+)	cobalt2	EX_cobalt2(e)	[e] : cobalt2 <==>	ion
chromate	cro4	EX_cro4(e)	[e] : cro4 <==>	ion
copper (1+)	cu	EX_cu(e)	[e] : cu <==>	ion
copper (2+)	cu2	EX_cu2(e)	[e] : cu2 <==>	ion
iron (2+)	fe2	EX_fe2(e)	[e] : fe2 <==>	ion
proton	h	EX_h(e)	[e] : h <==>	ion
water	h2o	EX_h2o(e)	[e] : h2o <==>	other
potassium	k	EX_k(e)	[e] : k <==>	ion
Magnesium (2+)	mg2	EX_mg2(e)	[e] : mg2 <==>	ion
manganese (2+)	mn2	EX_mn2(e)	[e] : mn2 <==>	ion
molybdate	mobd	EX_mobd(e)	[e] : mobd <==>	ion
nitrogen	n2	EX_n2(e)	[e] : n2 <==>	N source
sodium	na1	EX_na1(e)	[e] : na1 <==>	ion
ammonium	nh4	EX_nh4(e)	[e] : nh4 <==>	N source
nickel	ni2	EX_ni2(e)	[e] : ni2 <==>	ion
phosphate	pi	EX_pi(e)	[e] : pi <==>	P source
sulfite	so3	EX_so3(e)	[e] : so3 <==>	S source
sulfate	so4	EX_so4(e)	[e] : so4 <==>	S source
tungstate	tungs	EX_tungs(e)	[e] : tungs <==>	ion
zinc	zn2	EX_zn2(e)	[e] : zn2 <==>	ion
