# Supplementary material for: Constraint-Based Modeling of Carbon Fixation and the Energetics of Electron Transfer in Geobacter metallireducens
Source: PLoS Comput Biol. 2014 Apr 24;10(4):e1003575. doi: 10.1371/journal.pcbi.1003575 (PMC3998878; doi:10.1371/journal.pcbi.1003575)
Supplement: Text S1 — Supplementary text and methods. (PDF) [file pcbi.1003575.s013.pdf]

Text S1

Constraint-based modeling of carbon fixation and the energetics of external electron transfer in *Geobacter metallireducens*

Supplementary Text

Genome-wide Reconstruction Process

One feature of *G. metallireducens* that distinguishes its metabolic network from those of other bacteria is the high instance of isozymes. Comparatively, the *G. metallireducens* GS-15 network contains 409 instances of isozymes with a total metabolic reconstruction size containing 987 genes whereas that for *E. coli* K-12 MG1655 [1] is 346 instances for 1366 included genes. This level of redundancy indicates that *G. metallireducens* is well equipped to deal potential loss of function in a given gene function, but also may point to the fact that the gene functions in *G. metallireducens* are less characterized than that of *E. coli*.

There are a number of noteworthy pathways that are not yet represented *in silico* in a network reconstruction which were included in iAF987:

a) Carbon-fixation pathways – The genome annotation for *G. metallireducens* includes the content which enables autotrophic growth with formate or an external electron donor. The pathways which are encoded in the *G. metallireducens* genome and reconstructed in iAF987 are the reductive citric acid (TCA) cycle [2] and the dicarboxylate–hydroxybutyrate cycle [3]. Key enzymes which enable the function of these pathways had been identified [4]. Key enzymes for the reductive TCA cycle include the 2-oxoglutarate synthase (abbreviated OOR2r in the reconstruction) and ATP-citrate lyase (ACITL) which enable the citric acid cycle to run in reverse. For the dicarboxylate–hydroxybutyrate cycle, the key enzyme is 4-hydroxybutyryl-CoA dehydratase. Figure 2 depicts the two carbon fixation cycles included in the iAF987 reconstruction.
b) Aromatic degradation via a membrane-bound benzoyl-CoA reductase – Benzoyl CoA reductase is the key enzyme for the degradation of 8 aromatic substrates for *G.* metallireducens. This enzyme catalyzes the ferredoxin dependent endergonic reduction of beznyol CoA to Cyclohexa-1,5-diene-1-carbonyl-CoA. The mechanism for activation of this endergonic reaction was previously thought to be ATP driven (Sun et al, 2009). However, recent experimental results indicate that this is not ATP dependent and rather likely to be driven by membrane potential [5]. Thus, a proton translocating reaction was added to the reconstruction for this step in metabolism. The proton translocation stoichiometry was determined by a thermodynamic analysis, similar to the ones adopted for the other ETS reactions. The $\Delta E_0'$ for this redox couple was calculated to be -340 mV. Based on the theoretical proton per electron stoichiometry [6], at least 3 protons are required to be translocated per electron to drive this reaction. A total of two electrons are transferred in the reaction, hence the reaction was appropriately modified and included as follows:

   bzcoa + fdxr-4:2 + 6 h[e] → ch15deccoa + fdxo-4:2 + 4h[c]

c) Anaerobic menaquinone biosynthesis – The alternative pathway for menaquinone biosyntheisis, the futalosine pathway [7] [8] has been identified in the genome and was included in the iAF987.
d) Iron-sulfur clusters and molybdenum cofactors – Iron-sulfur clusters are essential cofactors for the function of many metalloproteins and several iron-sulfur cluster-binding proteins (e.g., nitrite reductase) are encoded in the *G. metallireducens* GS-15 genome [9]. Similarly, molybdenum is an important cofactor for the function of proteins [10]. As such, these pathways were included in the reconstruction of *G. metallireducens* GS-15 iAF987 and also the recently reconstructed *E. coli* K-12 MG1655 reconstruction concurrently.
e) LPS biosynthesis – Some Gram-negative bacteria contain 2,3-diaminino linkages for acyl-chains in their LPS molecules [11]. The genes encoding for this variant were determined to be present in the *G. metallireducens* genome (*G. metallireducens*et_2351 (gnnA) and *G. metallireducens*et_2352 (gnnB)), thus they were reconstructed into the network.

When comparing the iAF987 reconstruction to other reconstructions, the ModelSEED reconstruction was found to have 114 unique genes that were not present in the *i*AF987 reconstruction. Of these, 86 genes were involved in macromolecular synthesis, DNA replication, and protein modifications that are beyond the scope of a metabolic network, and 8 of them did not have a specific reaction association in the ModelSEED (i.e., generic terms such as aminopeptidase, amidohydrolase). Of the remaining 20 genes, only two (Gmet_0988 and Gmet_2683) were added to the reconstruction as isozymes for existing reactions; the other 18 assignments conflicted with our functional annotation of the genome and thus were not included. The specific references that were used to determine the content for these novel pathways included in the iAF987 reconstruction, as well as those which were used for the any other content in the network are included in Dataset S2.

To determine the unique reactions contained in the iAF987 reconstruction, it was compared to the following organisms and the respective reconstruction from the UCSD Systems Biology Research Group database. Most of the content is also accessible via the BiGG database (http://bigg.ucsd.edu, [12]): *Escherichia coli* [13,14], *Saccharomyces cerevisiae* [15,16], *Methanosarcina barkeri* [17], *Helicobacter pylori* [18], *Staphylococcus aureus* [19], *Yersinia pestis* [20], *Klebsiella pneumoniae* [21], *Mycobacterium tuberculosis* [22], Mitochondria [23], Human Recon 1 [24], *Pseudomonas putida* [25], and *Thermotoga maritima* [26].

The reconstruction presented here, iAF987, is a *G. metallireducens*-specific reconstruction. The name of the reconstruction was retained from a complementary study [27] as the same “base reconstruction” was utilized. The critical differences in the presented reconstruction and the co-culture model from the complementary study [27] are as follows:

1. The cost for extracellular electron transfer in the presented reconstruction and model for the entire electron transport system (ETS), the most common mode of growth for the organism, is not contained in the combined aforementioned co-culture model. This content is necessary for accurate modeling of single species behavior.
2. The presented reconstruction has an updated organism-specific GAM and NGAM based on pure culture experiments and modeling using *G. metallireducens*.
3. The aforementioned study only contains an integrated model, not a single species reconstruction. No analysis of *G. metallireducens* is given in that work.
4. Critical aspects and new content related to carbon dioxide fixation and electron flow are unique to this manuscript and represent a key finding.

Hence, both the aforementioned paper [27] and the *G. metallireducens* modeling presented in this study are in fact complementary to each other, by providing a detailed characterization of the metabolic physiology and energetics while in a co-culture, as well as single-species, growth via Fe(III) respiration or fumarate reduction.

Analysis of Transcriptomic Data using iAF987

Transcriptomics data from acetate to benzene was generated and analyzed with the iAF987 model. Of the 130 differentially expressed genes, 77 genes were up-regulated and 53 were down-regulated. The MADE algorithm predicted that during this shift, the expression of 885 genes in iAF987 do not change significantly and 102 genes are differentially expressed. Of the 102 differentially expressed genes, the MADE algorithm predicted the up-regulation of 70 genes and down-regulation of 32 genes. The model-based prediction of change in expression disagreed with the *in vitro* transcriptomic data for only 28 of the 987 genes leading to 97% overall agreement (Table S1). Of the 28 genes where model predictions are conflicted with experimental data, 7 genes are conflicted for upregulation and 21 for downregulation. Specifically, the experimental data showed upregulation of 77 genes, whereas model indicated the upregulation of only 70 genes. Similarly, while the data indicates the downregulation of 53 genes, the model indicated the downregulation of only 32 genes. Among the genes differentially expressed during the shift, the genes encoding for benzoyl-CoA reductase were up-regulated over 100-fold during benzoate growth. It was determined that this key enzyme that links the degradation of aromatic substrates to central metabolism is not ATP driven as previously thought [28], but is likely membrane bound and proton translocating [5]. Thus, a proton translocating reaction was added to the reconstruction for this step in metabolism. A translocation stoichiometry of 3 protons per electron was determined to be the likely extent of coupling through a thermodynamic analysis (see below).

Electron Transport System Reconstruction

The electron transport system of *G. metallireducens* can generally be divided into three parts. First, the electrons in the form of reducing equivalents derived during oxidation of the electron donor by the TCA cycle are transferred to the menaquinone pool at the inner membrane. Subsequently, the electrons from the menaquinone pool are transferred to the cytochromes at the inner-membrane. *Geobacter* then utilizes a series of periplasmic cytochromes to transfer these electrons to the outer-membrane cytochromes onto the terminal extracellular electron acceptor. In the case of the *dcuB* strain, where the terminal electron acceptor is fumarate, the electrons from the menaquinone pool are diverted towards fumarate reduction.

To reconstruct the electron transport system of *G. metallireducens*, all the key steps involving electron transfer to the terminal electron acceptor in these three phases were subject to a thermodynamic analysis. Specifically, the feasibility of proton translocation and the theoretical maximum proton translocation stoichiometry was determined using the equations described in [[6,29].

Transfer of reducing equivalents to menaquinone pool

NADH Dehydrogenase (reaction abbreviation, NADH17pp): There are two different potential NADH dehydrogenase complexes encoded in the *G. metallireducens* GS-15 genome (i.e., Nuo1 and Nuo2). Both of these complexes were assigned a functionality of translocating a net 3 protons out of the cell per 2 electrons tansfered from NADH to menaquinone. No organism-specific characterization of these enzymes has been performed. Therefore, the net 3 protons pumped out of the cell across the inner membrane per 2 electrons transferred was assumed via homology to other bacterial NADH dehydorgenases, such as that for *Escherichia coli* K12-MG1655 [30].

Fumarate Reductase (reaction abbreviation, FRD2rpp): Oxidation of succinate to fumarate in the TCA cycle plays a critical role in transferring electrons to the menaquinone pool. In *Geobacter* sp., this metabolic step is catalyzed by a bifunctional fumarate reductase/succinate dehydrogenase (FrdCAB) enzyme. While the redox coupling for fumarate reduction using menaquinol is energetically favourable, the oxidation of succinate using menaquinone is endergonic. This is primarily due to the fact that succinate/fumarate redox couple is more electropositive than the electron acceptor menaquinone. It has also been proposed that this apparent energetic cost for succinate oxidation could provide an explanation to the decreased growth rates observed for *G. sulfurreducens* while respiring on Fe(III) compared to fumarate reduction [31]. Moreover, initial studies in *B. subtilis* indicated the possibility of a transmembrane proton potential acting as the driving force for such a reaction [32]. The previous *Geobacter* models do not account for this based on biochemical evidence in organisms such as *Wolinella succinogenes* or *Rhodothermus marinus*.

However, more recent evidence indicates that succinate dehydrogenase catalyzed by the bifunctional FrdCAB does operate in a reverse redox loop in *D. vulgaris* [33]. This study shows that succinate oxidation by menaquinol requires the dissipation of membrane potential as a driving force. Furthermore, they attribute this coupling nature to the absence of the ‘uncoupling’ residue in FrdCAB of *W. succinogenes*. The presence of this residue is shown to dissipate the proton potential generated by fumarate reductase. Geobacter species also lack this residue. Hence we propose that the fumarate reductase of Geobacter species is also electrogenic. This implies that *Geobacter* species have an additional energy conserving step while respiring on fumarate. We evaluated this hypothesis by simulating fumarate respiration of the dcuB strain of *G. metallireducens* using the genome-scale model. The earlier version of the *G. metallireducens* model (iJS787) and the model (iAF987) without electrogenic fumarate reductase were unable to produce a feasible solution when constrained with experimentally measured acetate and fumarate uptake rates (see Table 3). However, the electrogenic fumarate reductase enabled the model to reproduce the experimental observations. Based on this evidence from the model and the biochemical evidence from *D. vulgaris*, we propose the incorporation of an electrogenic fumarate reductase in the Geobacter models.

Electron transfer from menaquinone pool to cytochromes across the membrane

The reaction CYTMQOR3pp denotes the transfer of electrons from the menaquinone pool to the cytochromes at the inner membrane. To evaluate the energetics of this reaction, the feasibility of proton translocation and the theoretical maximum proton translocation stoichiometry were determined [6]. Assuming the midpoint potentials for the cytochromes involved range from 136-155mV, the $\Delta E_0'$ was calculated to range between 210-229 mV. Consequently the maximum proton translocation stoichiometry was estimated to be between 1.5 to 1.75 per electron. Similar to the NADH dehydrogenase reaction, the stoichiometry of this reaction was modified to a net 3 protons translocated per 2 electrons transferred from the menaquinone pool to the cytochromes at the inner membrane.

Reactions PPCCYTCpp and OMCPPCOpp represent the transfer of electrons through the periplasmic cytochromes to the outer-membrane cytochromes of *G. metallireducens*. The reaction PPCCYTCpp denotes the electron transfer between periplasmic cytochromes (for eg. MacA and PpcA) where the electrons that were derived at the inner-membrane from the menaquinone pool are passed on to the periplasmic cytochromes. The reaction OMCPPCOpp represents the subsequent transfer of these electrons to the higher-potential outer-membrane cytochromes that are involved in Fe(III) reduction.

Reduction of Extracellular Fe(III)

It has been shown that *Geobacter* species reduce Fe(III) at its outer-membrane by transferring electrons from the outer-membrane cytochromes [34]. Hence, the reaction FERCYT has been included to represent electrons being transferred from reduced high-potential outer-membrane cytochromes to Fe(III)

ATPS10rpp (ATP synthase): The number of protons pumped across the cytoplasmic membrane per 3 ATP generated by the ATP synthase reaction was modified using growth yields obtained in a closely related species (*P. carbinolicus*) on ethanol and hydrogen [35]. Based on the yield data, a stoichiometry of 10 protons per 3 ATP was assigned to the ATP synthase reaction.

Dicarboxylate exchanger (dcuB) for modeling fumarate reduction in the dcuB strain

The reaction SUCFUMtpp (succinate/fumarate antiporter) represents the dicarboxylate exchanger *dcuB* where fumarate and succinate are exchanged in a stoichiometric ratio of 1:1. For every mole of fumarate transported into the cell from the periplasm, one mole of succinate is exported out of the cell into the periplasm. This reaction is the terminal reaction during fumarate reduction by the *dcuB* strain [31].

Functional Testing

Initial functional testing of the *G. metallireducens* network iAF987 was performed using a core biomass objective function, a well-known media condition which supports growth, and gap filling aided by the SMILEY algorithm [36]. The core biomass objective function was initially used to determine if the network could generate the necessary biomass components to replicate when growing on ferric citrate medium with acetate as an electron donor and carbon source. After the initial reconstruction phase of generating a 3-compartment curated *G. metallireducens* reconstruction, 9 of the 68 metabolites in the core BOF could not be generated. Therefore, the reconstruction was reviewed and further curated to investigate these deficiencies. Using inference based on pathway function, as well as the SMILEY computational algorithm [36] which predicts reactions which fills gaps in a metabolic network, reaction content was added to the network so that it could produce the necessary biomass components. For each biomass component which initially could not be generated, one reaction addition resolved production in each case. Specifically, of the nine metabolites, two of the compounds were metal ions necessary and transport reactions for these were added for a cation metal transporter gene. Six of the nine non-produced metabolites were cofactors or vitamins and the SMILEY algorithm aided in the identification of reactions which enabled production in two cases. The last component was a murein structure metabolite and a byproduct form its synthesis required reuptake for the reaction to be utilized in simulation, therefore a transporter was added in this case. This process was repeated using the wild-

type BOF (after the core biomass component gaps were filled) and 5 out of the 102 metabolites could not be generated. The addition of two different transporters to the network resolved these non-production issues. These transporter reactions uptake byproducts from the synthesis of five wild-type biomass components in the periplasm (murein and membrane lipid compounds). In total, 6 out of the 10 total reactions that were added to the network to fill gaps for the generation of biomass components were transport reactions. This result points to the poor characterization of transport genes, proteins, and specificity in microbial physiology. The complete list of biomass components that could not be generated initially and the reactions that were added to the network as a result of these deficiencies are given in Dataset S3.

Growth simulations using the reconstructed network, the wild-type BOF, and FBA were used to validate that the *G. metallireducens* reconstruction could simulate growth under 19 known growth conditions. It was previously identified that *G. metallireducens* could grow on 19 different carbon sources / electron donors while respiring on Fe(III) [37]. Using an estimated uptake rate similar to those determined in growth screens (see Table 3), it was confirmed that the simulations reproduced the ability of the *G. metallireducens* network to produce biomass for each of these conditions. This finding demonstrates that the network has sufficient coverage to examine *G. metallireducens* in all currently known growth conditions [37]. Further, the previous reconstruction can only be used to examine growth of *G. metallireducens* under 9 of these 19 known substrates. Extending this analysis further, we computed the additional growth substrates that have not been previously shown as sole carbon sources and electron donors and these are given in Table 1. After this functional testing, we turned our attention to modeling growth of *G. metallireducens* under various growth conditions.

Analysis of growth on additional substrates for the *dcuB* and wild type strains

In addition to analyzing the capabilities of *G. metallireducens* GS-15 under optimal growth conditions with acetate as the electron donor [38], growth capabilities were also analyzed when utilizing different electron donors. Comparison of *in silico* performance to experimental measurements revealed suboptimal performance of the *dcuB* strain grown with ethanol and butanol as electron donors (Table S2). This is in contrast to what was predicted when acetate was the electron donor. Simulations revealed that although the predicted optimal *in silico* acceptor to donor ratio was approximately the same as the experimental values (within the measured ratio including standard deviation), the resulting growth rates seen experimentally were much lower. This result indicates that there is more energy is being utilized for non-growth related activities when grown on ethanol and butanol, as compared to acetate. Furthermore, the standard deviations of the experimental measurements under these conditions are larger as butanol and ethanol were more difficult to measure accurately (likely due to their volatility).

References

1. Orth JD, Conrad TM, Na J, Lerman JA, Nam H, et al. (2011) A comprehensive genome-scale reconstruction of Escherichia coli metabolism - 2011. Mol Syst Biol 7.
2. Evans MC, Buchanan BB, Arnon DI (1966) A new ferredoxin-dependent carbon reduction cycle in a photosynthetic bacterium. Proceedings of the National Academy of Sciences of the United States of America 55: 928-934.
3. Huber H, Gallenberger M, Jahn U, Eylert E, Berg IA, et al. (2008) A dicarboxylate/4-hydroxybutyrate autotrophic carbon assimilation cycle in the hyperthermophilic Archaeum Ignicoccus hospitalis. Proceedings of the National Academy of Sciences of the United States of America 105: 7851-7856.
4. Berg IA, Kockelkorn D, Ramos-Vera WH, Say RF, Zarzycki J, et al. (2010) Autotrophic carbon fixation in archaea. Nature reviews Microbiology 8: 447-460.
5. Kung JW, Loffler C, Dorner K, Heintz D, Gallien S, et al. (2009) Identification and characterization of the tungsten-containing class of benzoyl-coenzyme A reductases. Proceedings of the National Academy of Sciences of the United States of America 106: 17687-17692.
6. Kroger A, Biel S, Simon J, Gross R, Unden G, et al. (2002) Fumarate respiration of Wolinella succinogenes: enzymology, energetics and coupling mechanism. Biochimica et biophysica acta 1553: 23-38.

7. Hiratsuka T, Furihata K, Ishikawa J, Yamashita H, Itoh N, et al. (2008) An alternative menaquinone biosynthetic pathway operating in microorganisms. Science 321: 1670-1673.
8. Arakawa C, Kuratsu M, Furihata K, Hiratsuka T, Itoh N, et al. (2011) Diversity of the early step of the futalosine pathway. Antimicrobial agents and chemotherapy 55: 913-916.
9. Aklujkar M, Krushkal J, DiBartolo G, Lapidus A, Land ML, et al. (2009) The genome sequence of Geobacter metallireducens: features of metabolism, physiology and regulation common and dissimilar to Geobacter sulfurreducens. BMC Microbiol 9: 109.
10. Schwarz G, Mendel RR, Ribbe MW (2009) Molybdenum cofactors, enzymes and pathways. Nature 460: 839-847.
11. Robins LI, Williams AH, Raetz CR (2009) Structural basis for the sugar nucleotide and acyl-chain selectivity of Leptospira interrogans LpxA. Biochemistry 48: 6191-6201.
12. Schellenberger J, Park JO, Conrad TM, Palsson BO (2010) BiGG: a Biochemical Genetic and Genomic knowledgebase of large scale metabolic reconstructions. BMC Bioinformatics 11: 213.
13. Feist AM, Henry CS, Reed JL, Krummenacker M, Joyce AR, et al. (2007) A genome-scale metabolic reconstruction for *Escherichia coli* K-12 MG1655 that accounts for 1260 ORFs and thermodynamic information. Mol Syst Biol 3.
14. Reed JL, Vo TD, Schilling CH, Palsson BO (2003) An expanded genome-scale model of *Escherichia coli* K-12 (*i*JR904 GSM/GPR). Genome Biology 4: R54.51-R54.12.
15. Duarte NC, Herrgard MJ, Palsson B (2004) Reconstruction and Validation of Saccharomyces cerevisiae iND750, a Fully Compartmentalized Genome-Scale Metabolic Model. Genome Res 14: 1298-1309.
16. Mo ML, Palsson BO, Herrgard MJ (2009) Connecting extracellular metabolomic measurements to intracellular flux states in yeast. BMC Syst Biol 3: 37.
17. Feist AM, Scholten JCM, Palsson BO, Brockman FJ, Ideker T (2006) Modeling methanogenesis with a genome-scale metabolic reconstruction of Methanosarcina barkeri. Mol Syst Biol 2: 1-14.
18. Thiele I, Vo TD, Price ND, Palsson B (2005) An Expanded Metabolic Reconstruction of Helicobacter pylori (*i*IT341 GSM/GPR): An *in silico* genome-scale characterization of single and double deletion mutants. J Bacteriol 187: 5818-5830.
19. Becker SA, Palsson BO (2005) Genome-scale reconstruction of the metabolic network in Staphylococcus aureus N315: an initial draft to the two-dimensional annotation. BMC Microbiol 5: 8.
20. Charusanti P, Chauhan S, McAteer K, Lerman JA, Hyduke DR, et al. (2011) An experimentally-supported genome-scale metabolic network reconstruction for Yersinia pestis CO92. BMC Syst Biol 5: 163.
21. Liao YC, Huang TW, Chen FC, Charusanti P, Hong JS, et al. (2011) An experimentally validated genome-scale metabolic reconstruction of Klebsiella pneumoniae MGH 78578, iYL1228. Journal of Bacteriology 193: 1710-1717.
22. Jamshidi N, Palsson BO (2007) Investigating the metabolic capabilities of Mycobacterium tuberculosis H37Rv using the in silico strain iNJ661 and proposing alternative drug targets. BMC Syst Biol 1: 26.
23. Vo TD, Greenberg HJ, Palsson BO (2004) Reconstruction and functional characterization of the human mitochondrial metabolic network based on proteomic and biochemical data. J Biol Chem 279: 39532-39540.
24. Duarte NC, Becker SA, Jamshidi N, Thiele I, Mo ML, et al. (2007) Global reconstruction of the human metabolic network based on genomic and bibliomic data. Proc Natl Acad Sci U S A 104: 1777-1782.
25. Nogales J, Palsson BO, Thiele I (2008) A genome-scale metabolic reconstruction of Pseudomonas putida KT2440: iJN746 as a cell factory. BMC Syst Biol 2: 79.
26. Zhang Y, Thiele I, Weekes D, Li Z, Jaroszewski L, et al. (2009) Three-dimensional structural view of the central metabolic network of Thermotoga maritima. Science 325: 1544-1549.
27. Nagarajan H, Embree M, Rotaru AE, Shrestha PM, Feist AM, et al. (2013) Characterization and modelling of interspecies electron transfer mechanisms and microbial community dynamics of a syntrophic association. Nature communications 4: 2809.
28. Sun J, Sayyar B, Butler JE, Pharkya P, Fahland TR, et al. (2009) Genome-scale constraint-based modeling of Geobacter metallireducens. BMC Syst Biol 3: 15.
29. Thauer RK, Jungermann K, Decker K (1977) Energy conservation in chemotrophic anaerobic bacteria. Bacteriological reviews 41: 100-180.

30. Noguchi Y, Nakai Y, Shimba N, Toyosaki H, Kawahara Y, et al. (2004) The energetic conversion competence of *Escherichia coli* during aerobic respiration studied by 31P NMR using a circulating fermentation system. J Biochem (Tokyo) 136: 509-515.
31. Butler JE, Glaven RH, Esteve-Nunez A, Nunez C, Shelobolina ES, et al. (2006) Genetic characterization of a single bifunctional enzyme for fumarate reduction and succinate oxidation in Geobacter sulfurreducens and engineering of fumarate reduction in Geobacter metallireducens. Journal of Bacteriology 188: 450-455.
32. Schirawski J, Unden G (1998) Menaquinone-dependent succinate dehydrogenase of bacteria catalyzes reversed electron transport driven by the proton potential. European journal of biochemistry / FEBS 257: 210-215.
33. Zaunmuller T, Kelly DJ, Glockner FO, Unden G (2006) Succinate dehydrogenase functioning by a reverse redox loop mechanism and fumarate reductase in sulphate-reducing bacteria. Microbiology 152: 2443-2453.
34. Inoue K, Qian X, Morgado L, Kim BC, Mester T, et al. (2010) Purification and characterization of OmcZ, an outer-surface, octaheme c-type cytochrome essential for optimal current production by Geobacter sulfurreducens. Applied and environmental microbiology 76: 3999-4007.
35. Haveman SA, DiDonato RJ, Jr., Villanueva L, Shelobolina ES, Postier BL, et al. (2008) Genome-wide gene expression patterns and growth requirements suggest that Pelobacter carbinolicus reduces Fe(III) indirectly via sulfide production. Applied and environmental microbiology 74: 4277-4284.
36. Reed JL, Patel TR, Chen KH, Joyce AR, Applebee MK, et al. (2006) Systems approach to refining genome annotation. Proceedings of the National Academy of Sciences of the United States of America 103: 17480-17484.
37. Lovley DR, Giovannoni SJ, White DC, Champine JE, Phillips EJ, et al. (1993) Geobacter metallireducens gen. nov. sp. nov., a microorganism capable of coupling the complete oxidation of organic compounds to the reduction of iron and other metals. Archives of microbiology 159: 336-344.
38. Lovley DR, Phillips EJ (1988) Novel mode of microbial energy metabolism: organic carbon oxidation coupled to dissimilatory reduction of iron or manganese. Applied and environmental microbiology 54: 1472-1480.
